# Strengthening health systems to provide rehabilitation services

**DOI:** 10.2471/BLT.17.191809

**Published:** 2017-03-01

**Authors:** Etienne Krug, Alarcos Cieza

**Affiliations:** a Department for Management of Noncommunicable Diseases, Disability, Violence and Injury Prevention, World Health Organization, Avenue Appia 20, 1211 Geneva 27, Switzerland.

The world faces new challenges in light of health and demographic trends: populations are ageing, and the number of people living with noncommunicable diseases and the consequences of injuries is increasing.^1^^–^^3^ The health, social and economic consequences of these trends should serve as a call to policy-makers to invest not only in health services that reduce mortality and morbidity, but also in those that improve functioning and consequently well-being. These latter outcomes are at the core of rehabilitation, yet rehabilitation services are often underdeveloped, underresourced and undervalued.

A dramatic increase in the absolute number of years lived with disability (YLDs) combined with a rising prevalence of severely disabling conditions have led to a demand for rehabilitation that is largely going unmet. Seventy-four per cent of YLDs are the result of health conditions for which rehabilitation may be beneficial.^4^ The prevalence of health conditions associated with severe levels of disability has increased by nearly 23% since 2005.^5^ Yet in many parts of the world, the size of the rehabilitation workforce is insufficient. For example, in the World Health Organization (WHO) African and Eastern Mediterranean Regions, the number of trained professionals required to meet the demand for rehabilitation services (such as occupational therapists, physiotherapists and speech therapists) is estimated to be a tenth of that required.^4^

Factors contributing to the unmet need for rehabilitation services include poor accessibility, transport barriers, high out-of-pocket expenses and long waiting times.^6^^,^^7^ An additional factor is a lack of awareness of the need for rehabilitation; what it is, what it does, and whom it may benefit. Rehabilitation comprises a set of interventions designed to reduce disability and to optimize functioning in individuals with health conditions so as to enable them to better interact with their environment. As such, it is not restricted to a minority group of persons with disabilities or those with significant long-term impairments. Rehabilitation is also relevant to people experiencing limitations in functioning associated with ageing, an injury or other conditions. For example, evidence indicates that rehabilitation improves functioning for those who have suffered acute myocardial infarction or strokes, while it can also have positive results for those with musculoskeletal or mental health conditions.^8^^–^^11^

The role that rehabilitation plays in maximising the impact of other health services – such as surgical interventions, trauma care and management of noncommunicable diseases – and its potential for significant cost savings are also frequently misunderstood and underestimated.^12^ For example, rehabilitation has been found to be beneficial in reducing length-of-stay in hospitals and decreasing re-admissions, thus mitigating the negative social and health risks associated with prolonged hospitalizations. In the context of complex conditions that require intensive and highly specialized rehabilitation, cost savings to both the individual and the health sector may be realized. By improving a person’s ability to participate more fully in everyday life, rehabilitation reduces the costs related to ongoing care and support and may accelerate the ability to return to education or employment.

Rehabilitation is part of universal health coverage and should be incorporated into the package of essential services, along with prevention, promotion, treatment and palliation. To this end, on 7 February 2017, WHO, Member States, international and professional organizations, nongovernmental organizations and rehabilitation experts issued *Rehabilitation 2030: a call for action,* a commitment to key actions to strengthen rehabilitation services in Member States.^4^ These actions include: improving rehabilitation governance and investment; expanding a high-quality rehabilitation workforce; and enhancing rehabilitation data collection. The commitment to strengthen health systems to provide rehabilitation services should make it possible for millions of people not only to live longer, but to live well.
